# Phthalate Exposure and Biomarkers of Oxidation of Nucleic Acids: Results on Couples Attending a Fertility Center

**DOI:** 10.3390/toxics10020061

**Published:** 2022-01-29

**Authors:** Daniela Pigini, Lidia Caporossi, Enrico Paci, Silvia Capanna, Paola Viganò, Alessandra Alteri, Elisa Rabellotti, Flavia Buonaurio, Bruno Papaleo, Giovanna Tranfo

**Affiliations:** 1 INAIL, Department of Occupational and Environmental Medicine, Epidemiology and Hygiene, Via Fontana Candida 1, 00078 Monte Porzio Catone, Italy; d.pigini@inail.it (D.P.); e.paci@inail.it (E.P.); s.capanna@inail.it (S.C.); b.papaleo@inail.it (B.P.); g.tranfo@inail.it (G.T.); 2 Obstetrics and Gynecology Unit, IRCCS San Raffaele Scientific Institute, Via Olgettina 60, 20132 Milan, Italy; pa.vigano@gmail.com (P.V.); alteri.alessandra@hsr.it (A.A.); rabellotti.elisa@hsr.it (E.R.); 3 School of Doctorate in Chemical Sciences, Department of Chemistry, Sapienza University of Rome, Piazza Aldo Moro 5, 00185 Roma, Italy; flavia.buonaurio@uniroma1.it

**Keywords:** phthalates, nucleic acids oxidation, nitroxidation, methylation, oxidative stress, urinary biomarkers, infertility

## Abstract

Phthalates are substances used as plasticizing agents and solvents that can increase the risk of infertility and that appear to induce oxidative stress. The aim of the study was to show the possible relationship between urinary concentrations of phthalates metabolites, namely MEP, MBzP, MnBP, MEHP, MEHHP, and MnOP and biomarkers of nucleic acids oxidation, methylation, or protein nitroxidation. The oxidative stress biomarkers measured in human urine were 8-oxo-7,8-dihydroguanine, 8-oxo-7,8-dihydroguanosine, 8-oxo-7,8-dihydro-2′-deoxyguanosine, 3-nitrotyrosine, and 5-methylcytidine. Two hundred and seventy-four couples were enrolled, undergoing an assisted reproduction technology (ART) treatment, urine samples were analyzed in HPLC/MS-MS, and then two sub-groups with urinary concentration > 90th or <10th percentile were identified, reducing the sample size to 112 subjects. The levels of oxidative stress biomarkers were measured in both groups, reduced to 52 men and 60 women. A statistically significantly difference for 8-oxoGuo and 3-NO_2_Tyr between men and women, with higher levels in men, was found. The levels of oxidative stress biomarkers were directly correlated with some phthalate concentrations in both sexes.

## 1. Introduction

Exposure to phthalate seems to increase oxidative stress levels, which is recognized to contribute to male and female infertility. Phthalates are substances that exhibit endocrine disrupting properties and can induce problems related to infertility, disorders of the metabolic, immune, and thyroid systems [1]. They are a family of esters of phthalic acid, widely used in industry as plasticizing agents, added to improve the flexibility and moldability of plastics and widely used in children’s toys, medical devices, cosmetics, hygiene products, clothing such as footwear and raincoats, food packaging, and also construction material [2]. The phthalates with lower molecular weight are highly soluble in water (i.e., diethyl phthalate (DEP) and di-n-butyl phthalate (DnBP)) and therefore they are inserted in production cycle of cosmetics, pharmaceuticals, and insecticides. The EU directive on cosmetics, Dir 76/768/EEC, prohibits in Europe the use of DnBP together with other phthalates such as (2-ethylhexyl)-phthalate (DEHP), benzyl butyl phthalate (BzBP), bis2-methoxyethyl phthalate, n-pentylisopentyl phthalate, di-n-pentyl phthalate, and diisopentyl phthalate.

DEHP, due to its toxicity for reproduction, has recently been replaced with di-isononyl phthalate (DiNP), also in the production of PVC [3]. Phthalates, at high concentration, have toxic effects on the liver and kidneys, and cause malformations and fetal death.

In men, they cause a decrease in concentrations of testosterone and consequently of sperm quality, since they act as xenoestrogens [3]. Some studies provide evidence that phthalate exposure may be associated with a decrease in fertility and fecundity [4,5].

Recent studies proposed a correlation between the phthalate metabolites and the oxidation products of nucleic acids [6]. In fact, the oxidative stress (OS) could be involved in the mechanisms by which phthalates cause adverse effects. Higher OS and greater difficulties of cells to deal with it can lead to affect the reproductive organs and reduce fertility [7]. It is well known that oxidative stress induced by xenobiotics can affect the male reproductive tract and spermatogenesis, which results in the production of sperm of poor quality [8,9]. Spermatozoa are unable to defend themselves effectively against oxidative stress [10]. The imbalance between pro-oxidants and antioxidants can lead to a number of reproductive diseases such as endometriosis, polycystic ovary syndrome (PCOS), and unexplained infertility. Pregnancy complications such as spontaneous abortion, recurrent pregnancy loss, and preeclampsia, can also develop in response to OS [11].

OS is an imbalance between the production of ROS (reactive oxygen species) and the ability of the biological system to repair the damage. In physiological processes, ROS could be key signal molecules from the growth of oocytes to conception, embryo development and pregnancy. ROS also play a role in the pathological processes affecting the female reproductive tract, influencing the entire reproductive life of women; moreover, the OS can modulate the decrease in fertility related to age [12].

Oxidative and nitrosative stress are closely related; ROS react with nitrogen species (RNS) when ROS levels are heightened. In addition, ROS and RNS can induce pathologic effects to reproduction function, acting on the mitochondrial and nuclear DNA, lipids, proteins, and spermatozoa biomolecules [13,14,15,16,17].

Urinary oxidized products of nucleic acids can be considered suitable biomarkers of oxidative stress. Among the nitrogenous bases contained in the nucleic acids, guanine has a lower redox potential and so it’s the most vulnerable to oxidation. The creation and excretion of the oxidized form 8-oxo-7,8-dihydroguanine (8-oxoGua) is the result of the activities of DNA reparation and of both DNA and RNA turnover.

The 8-oxodGuo (8-oxo-7,8-dihydro-2-deoxyguanosine) (8-oxodGuo) molecule is formed by the bound of guanine with deoxyribose, obtaining the oxidized nucleoside deoxy-guanosine, and it is the product of an important DNA lesion coming from repair and turnover, while the bind between guanine and ribose molecule produces the 8-oxoGuo (8-Oxo-7,8-guanosine) (8-oxoGuo) that is the oxidized nucleoside guanosine, produced by RNA turnover. The molecular characteristics of RNA (single-stranded) bring a higher easiness of ROS attack than DNA because its bases are sterically less protected [18,19].

The nitration of tyrosine residues is the result of greater RNS levels, which is realized in two steps: first, the production of tyrosyl radicals, and second, with their reaction with NO2 obtaining the formation of 3-nitrotyrosine (3-NO2Tyr) [12].

DNA methylation plays vital roles in numerous biological processes: Numerous types of methylated nucleosides were identified, which modulate the functions of RNA. 5-MeCyt (5-methylcytidine) (5-MeCyt) is a methylation product of cytidine, the nucleoside formed by cytosine bounded to ribose, and this molecule is used as an epigenetic biomarker for RNA, because it impacts on its structure and functions [20].

The aim of the study herein presented was to evaluate the relationship between phthalate metabolites levels and biomarkers of nucleic acids oxidation and protein nitroxidation in the urine of male and female subjects attending a fertility center. The main objective was to understand if these chemicals were able to induce oxidative stress and if this pathway could be responsible for some infertility problems.

## 2. Materials and Methods

### 2.1. Study Population

The Institutional Review Board of the IRCCS San Raffaele Scientific Institute in Milan evaluated and expressed a positive opinion about the study protocol (identification code 73/INT/2017, date 8 June 2017).

A total of 274 couples undergoing an assisted reproduction technology (ART) treatment at the San Raffaele Hospital (Milan, Italy) were enrolled.

All the subjects were interviewed, following the signing of the informed consent.

Anamnestic information was collected using an ad hoc questionnaire, and in addition to the clinical history, data were gathered about life habits and occupational activity.

The analysis of urinary phthalates metabolites was carried out for all the participants and the results obtained have been previously published [21,22]. In these studies the analytes were: MEP (monoethyl phthalate), metabolite of DEP; MBzP (monobenzyl phthalate), metabolite of BzBP; MnBP (mono-n-butyl phthalate), metabolite of both DnBP and BBzP; MEHP (mono-(2-ethylhexyl)phthalate) and MEHHP (mono-2-ethyl-5-hydroxyhexylphthalate), metabolites of DEHP; MnOP (mono-n-octyl phthalate) metabolite of DnOP (di-n-octyl phthalate).

Subsequently, from these data, for a better underlining of the differences, two sub-groups were identified based on a urinary concentration > 90th or <10th percentile for at least one of the studied metabolites. These two groups were selected for the determination of the urinary level of 8-oxoGua, 8-oxoGuo, 8-oxodGuo, 3-NO_2_Tyr, and 5-MeCyt.

This selection led to a final group of 112 subjects.

In Table 1 the number of subjects having high (>90th percentile) or low (<10th percentile) levels of each considered phthalate metabolite is reported. Overall, the sample selected for the determination of oxidative stress biomarkers was reduced to 52 men and 60 women.

### 2.2. Urine Sample Collection

Methods for collection of urine samples have been previously described [21]; a spot volume of urine was collected during the outpatient visits of the recruited subjects, cases, and controls. One aliquot (250 µL) was used for the determination of the oxidative stress biomarkers: 8-oxoGua, 8-oxoGuo, 8-oxodGuo, 3-NO_2_Tyr, and 5-MeCyt, another (3 mL) for phthalate metabolites analysis.

### 2.3. Biological Monitoring

The concentrations of the six phthalate metabolites MEP, MBzP, MnBP, MEHP, MEHHP, and MnOP were determined as previously described [4].

Briefly, urine samples were purified with SPE extraction and analyzed with the HPLC/MS/MS system (API-4000, ABI Sciex, Framingham, MA, USA), using a Synergi Polar 150 × 4.6-mm column and an acetic acid 1% *v*/*v* solution in water/acetonitrile gradient as mobile phase. The method was fully validated with accuracy between 91.89% and 100.65%, an interday variability between 1.8% and 7.8%, and limit of detection (LOD) from 0.05 µg/L (MEHHP) to 3 µg/L (MEP).

The analytical method used for the 8-oxoGua, 8-oxoGuo, 8-oxodGuo, and 3-NO2Tyr determination was the one published by Andreoli et al. [23] with some changes; the chromatographic column used was a Kinetex Polar C18 column 100 A (150 × 4.6 mm, 2.6 m) supplied by Phenomenex (Torrance, CA, USA); for 5-MeCyt the same method was followed, after diluting the sample 1:100 and using a chromatographic column Discovery C18 (150 × 4.6 mm, 5 µm) by Supelco Analytical, Bellefonte, PA, USA).

Sample pretreatment provided a defrost at 37 °C and a subsequent centrifugation at 10,000× *g* for 5 min, and then the internal standard was added to the urine supernatant and it was injected into the HPLC-MS/MS system.

The monitored ionic transitions were studied in the positive ion mode and were (precursor/product values of *m*/*z*): 168.0→140.0 and 171.0→143.0 for 8-oxoGua and its internal standard ((^13^C^15^N_2_) 8-oxoGua), 284.3→168.0 and 287.13→171.1 for 8-oxodGuo and its internal standard ((^13^C^15^N_2_) 8-oxodGuo), 300.24→168.2 and 303.24→171.0 for 8-oxoGuo and its internal standard ((^13^C^15^N_2_) 8-oxoGuo), 226.99→181.0 and 229.99→184.0 for 3-NO_2_Tyr and its internal standard (3-NO_2_Tyr-d_3_), 257.95→126.100 and 180.3→80.10 for 5-MeCyt and Cotinine d_3_ used as internal standard, respectively. The LODs were: 0.50 µg/L for 8-oxoGua, 0.14 µg/L for 8-oxodGuo,0.70 µg/L for 8-oxoGuo, 1.81 µg/L for 3-NO2Tyr, and 0.28 µg/L for 5-MeCyt.

The chromatographic runs were carried out with a Series 200 LC quaternary pump (PerkinElmer, Norwalk, CT, USA) coupled with an AB/Sciex API 4000 triple-quadrupole mass spectrometry detector equipped with a Turbo Ion Spray (TIS) probe. Detection was in the MRM mode. The used instrument software was 1.5 version of Analyst^®^ software (AB Sciex, Framingham, MA, USA).

To normalize the values of analytes urinary concentrations, owing to dilution, the creatinuria was measured, using the method of Jaffè [24], and the concentration values of the analytes were expressed in µg/g of creatinine.

### 2.4. Statistical Analysis

For the statistical elaboration the software SPSS^®^ 25.0 (IBM, Armonk, NY, USA) was used.

Firstly, a descriptive analysis was produced to draw a general picture of the study population; for the male–female comparison, the χ^2^ test was used for qualitative variables and the *t*-test for quantitative variables, both with a significance level of 0.05.

Values of the bases do not have a normal distribution; consequently, data were logarithmic (log 10) transformed to obtain it. Values of phthalate metabolites or of oxidative bases below the limit of detection (LOD) were set to LOD/2.

Multiple linear regressions were performed to hypothesize a possible cause–effect relationship between each base, and the values of the analyzed phthalates, MnBP, MEP, MBzP, MnOP, and ∑DEHP, were considered as independent variables. The regressions were conducted for the whole sample and then separately for men and women. In the regression study, age, BMI, smoke habit, and alcohol consumption were taken into consideration as confounding factors.

Finally, a student’s *t*-test was performed to highlight the differences in the mean values of the oxidative stress biomarkers depending on the percentile group for the phthalate metabolites concentration. A significance level of 0.05 was chosen as useful.

## 3. Results

All results are herein presented in tables. Table 2 summarizes the sample characteristics of recruited subjects. Table 3 and Table 4 show the urinary phthalate metabolites concentrations for the selected subjects, while Table 5 highlights the results regarding the oxidative stress biomarkers.

The comparison between mean values of men, reported in Table 5, and those of women, reported in Table 6, showed a statistically significantly difference for 8-oxoGuo (*p* = 0.013) and 3-NO_2_Tyr (*p* = 0.002), and both levels resulted higher in men.

Multiple linear regressions were performed (Table S1) to evaluate possible correlations between oxidative stress biomarkers and phthalate metabolites levels, considered as independent variables. In the male sample the greater significance was found between the MBzP and 8-oxoGua (*p* = 0.011) and between MBzP and 5-MeCyt (*p* = 0.035). Instead, in the female sample, a significant correlation emerged between MEP and 8-oxoGua (*p* = 0.019) and between MnOP and 8-oxodGuo (*p* = 0.031).

A Student’s *t*-test was performed to highlight differences in oxidative stress biomarkers’ mean values (log-transformed) depending on the percentile group (>90th or <10th percentile). A level of significance of 0.05 was chosen and significant findings were reported in Table 7 for the two groups.

The results showed higher levels of oxidative stress biomarkers for higher concentration of phthalate metabolites, confirmed in both groups.

## 4. Discussion

The consideration of sex as a specific discriminant of possible health effects and the subsequent definition of the gender specific medicine is an important step in the human knowledge [25].

Gender-related biological differences might stand out in different susceptibility of males and females to risk factors as, for instance, exposure to hazardous substances or biological agents [26,27,28]. For this reason, in our study male and female data were analyzed separately, also taking into account that phthalates are estrogen-like substances, and therefore, different effects on males and females were expected [29]. Moreover, the two groups of subjects showed significant differences in body mass index (BMI), alcohol consumption, and working activity.

Signs of the presence of oxidative stress consist in the oxidative damage of DNA, RNA, lipid, and protein [30] due to the increased levels of ROS, peroxides, and other oxygen species.

Higher levels of ROS were linked to different reproductive health problems, starting from a lower follicle quality [31], endometriosis [7], up to a role in infertility, with documented negative interference in assisted reproductive techniques success [7,32]. Researchers also showed that higher ROS levels were associated with the initiation of apoptosis in antral follicles [33].

It was suggested that the oxidative stress could be an intermediate mechanism to obtain endocrine dysfunction due to endocrine disrupting chemicals, so that some authors proposed to use the evaluation of ROS as a possible early marker of toxicity [34,35]. The pathway used by endocrine disruptors to induce oxidative stress in reproductive tissue seems to be an interference in antioxidant protective mechanism [36,37].

To go into more detail, a review was proposed by Asghari et al. [38] to understand the biochemical mechanisms of toxicity of phthalates, in particular for reproductive organs, and some evidence emerged about the involvement of generation of ROS and the subsequent DNA damage, lipid peroxidation, and an interference with the activity of important antioxidant enzymes. Even with some controversy, it seems that a reduction of superoxide dismutase and an increase of catalase is commonly observed with higher phthalate exposure; furthermore, glutathione peroxidase and glutathione S-transferase activities are decreased. In reproductive organs these biochemical changes, linked to OS, could lead to a reduction of spermatogenesis, dysfunction in gonocytes, and an increase of peroxiredoxin 3 and cyclooxygenase 2 concentrations in spermatocytes.

The scientific literature includes studies on the correlation of phthalates exposure with DNA damage, in fertile women more than in men [5,39,40,41], in child populations [6], in adolescents and adults [42], in couples planning for pregnancy [5], and in pregnant women [5,6]. In these studies, 8-oxo-dGuo was the only biomarker evaluated. In particular, higher values of 8-oxo-dGuo were found linked to higher phthalate exposure with regard to DEHP, DnBP, and DiBP [5,43,44]. In Table S2 some results are summarized some about these published papers.

To understand the association between phthalates metabolites and DNA damage it was observed that changes occurred in the metabolism of lipids, due to peroxidation, with many differences during the pregnancy, and it was suggested that an induction of reactive oxygen/nitrogen species (ROS/RNS), due to phthalates, could be an intermediate event in possible adverse outcomes [45].

8-oxo-dGuo is the most used oxidative stress biomarker [39,41,46], and in numerous surveys it is usually the only analyte determined for the purpose. Given the absence of data about other pathways, obtainable information about the whole oxidative stress level is limited. In the present study, we chose to also analyze an RNA oxidation biomarker, a nitration biomarker, and a methylation biomarker, to provide a more complete overview.

Our results showed that 8-oxoGuo is the most sensible short-term biomarker of oxidative stress and reversible exposure to chemicals. Furthermore, the correlation with 5-MeCyt confirms the greater susceptibility of RNA [47] in which the guanine, being RNA a single-strand macromolecule, is less protected from oxidation compared to DNA where, the presence of the double strand can sterically shield the basis. Moreover, differently from what happens in DNA, there are no repair mechanisms for damaged RNA.

Regarding the analytical methods, several studies used an ELISA, instead of more sensitive and accurate methods, such as HPLC-MS/MS. The choice of the method could bring to possible analytical bias, namely low quantification limits, low selectivity, and interferences [46,48,49,50,51]. Therefore, we decided to use an HPLC/MS/MS procedure to minimize the analytical error.

The comparison between the different groups of subjects, with “high” and “low” phthalates metabolites concentrations showed relevant differences in oxidative stress biomarkers.

8-oxoGua did not show a significant increase in high phthalates exposed subjects, but no information can be found in the literature about biomonitoring of this biomarker related to phthalate exposure.

On the contrary, men with high values of DEHP metabolites, concentrations showed a significant correlation with four oxidative stress biomarkers, namely 8-oxoGuo, 8-oxodGuo, 3 NO2Tyr, and 5-MeCyt; the same situation was found in women for three biomarkers, 8-oxoGuo, 3-NO2Tyr, and 5-Mecyt.

Phthalate esters are a family of chemicals whose structure only varies in the length of their ortho-positioned hydrocarbon chain ‘arms’. Chain length varies from 1 to >10 carbons, in linear or branched format. This different molecular structure brings different possible toxicological profiles. DEHP in Europe is under a restriction regime, following the release of the European Regulation (UE) 1906/2006, and since 7 July 2020, the manufacture, market, or use of this phthalate have been banned. The decision was based on the toxicological profile of DEHP, particularly detrimental on the reproductive sphere. Our findings confirmed that DEHP could be considered one of the most toxic phthalates [52].

In our study, MBzP showed a significant relation with 8-oxo-Guo, both in men and women. However, in men, it was also linked with 3-NO2Tyr, and only in women with 5-MeCyt. To the best of our knowledge, no other previously published study proposed this type of link. MnBP in women was significantly related with 8-oxoGuo, 3-NO2Tyr, and 5-MeCyt, while in men, only with 3-NO2Tyr. As a metabolite of DnBP, a lot of evidence is reported in the literature about its ability to harm the embryonal development, mediated by the oxidative stress due to ROS increase [53]. In both men and women, MnOP showed a significant correlation with 5-MeCyt. In fact, DnOP exposure, of which MnOP is the metabolite, is related to reproductive toxicity, because of the oxidative stress caused by the proliferation of peroxisomes, and this should explain our results on this phthalate metabolite [54].

Lastly, MEP was well correlated in women with 8-oxoGuo, while in men the significant correlation was with 5-MeCyt. We should here underline the fact that DEP is not subject to any restriction, as it is not yet included in the authorization list of European Regulation (UE) 1906/2006. It is therefore extensively present in daily products, such as creams and makeups or personal hygiene products, even in large amounts, as shown in biological monitoring studies [3]. This may explain why researchers find a greater presence of MEP in the female subjects. Differences between the two groups (high and low) were more frequently demonstrated for 5-MeCyt, followed by 8-oxoGuo and by 3-NO2Tyr. While DEP effects are not widely studied yet in literature, some authors supported some DNA damage impairing semen functions [55,56].

Poor correlations were observed for 8-oxodGuo; these data suggest a weak association of phthalates levels with the DNA damage compared to damage to RNA and proteins.

The present study has limitations related to the specificities of the sampling conditions and to its own characteristics. In detail they can be identified as:

- The need to proceed with spot urine sampling, due to the impossibility of knowing the exact moment of exposure to phthalates does not allow to identify the best an exact sampling timing that allows to take into account the half-time of each compound. This could lead to an underestimation of the exposure level;
- The biomonitoring data collected do not properly represent the “biologically effective dose”, which is the concentration of substance that reaches the target organ, in this specific case the reproductive system, even if these results allow to obtain information on exposure. Nevertheless, considering that the oxidative stress data are also determined in the urinary matrix and not in specific cells of the target organ (i.e., in seminal liquid cells) and therefore it is an information linked with the whole organism, the consideration of urinary levels of phthalates seems to be an appropriate choice;
- The metabolism of phthalates involves a series of enzymes, knowing (i.e., through single nucleotide polymorphism analysis) the characteristics of which would help to evaluate possible differences in metabolism speed that, in certain measure could affect the toxic effects recorded, also with the same exposure to the toxic. This biochemical investigation was not conducted, considering the impossibility of identifying a specific time of exposure to the toxics for the recruited subjects, as considerations regarding the rate of metabolism and excretion could not lead to specific observations. The lack of such information could lead to an underestimation of the effects;
- The small number of subjects in the two groups of population having high and low values of exposure to phthalates represents a limitation in the extrapolation of the data to a more general situation. Further investigation with more numerous samples could confirm our findings with greater strength, and perhaps could support greater differences between genders.

## 5. Conclusions

In our study, the urinary concentration levels of 8-oxoGuo and 3-NO_2_Tyr were higher in men than in women, while the concentration levels of the other indicators did not differ between genders. This makes it possible to highlight the different vulnerability between men and women to the exposure to dangerous substances.

This study also highlighted the possible correlation among phthalates exposure and oxidative stress, in an infertile population.

Our findings permit us to focus on DEHP as the most toxic phthalate, as expected, but also underline that other phthalates are involved, in different ways, in oxidative damage. At the same time, 8-oxoGuo emerged as the most sensitive and susceptible biomarker of oxidative stress, for short-term exposure to chemicals, confirming an attack of ROS and RNS on RNA. In addition, considerations about other biomarkers such as 8-oxodGuo, 5-MeCyt, and 3-NO_2_Tyr must be investigated, owing to their correlation with different phthalates.

The results of biological monitoring of phthalates exposure showed statistically significant correlations with the biomarkers of oxidative stress, 8-oxoGuo, 8-oxodGuo, 3-NO_2_Tyr, and 5-MeCyt, confirming that exposure to phthalates of various origins may induce oxidative stress, and, to some extent, it could suggest an involvement in the reproductive system problems.

## Figures and Tables

**Table 1:** Number of subjects with urinary phthalate metabolite concentration over 90th percentile or <10th percentile of the group.

Phthalate	N > 90th (M/W ^f^)	N < 10th (M/W)
MnBP ^a^	25 (12/13)	21 (7/14)
MEP ^b^	17 (9/8)	24 (15/9)
MBzP ^c^	23 (9/14)	29 (10/19)
MnOP ^d^	22 (12/10)	28 (11/17)
∑DEHP ^e^	28 (14/14)	16 (4/12)

^a^ MnBP—mono-n-butyl phthalate; ^b^ MEP—monoethyl phthalate; ^c^ MBzP—monobenzyl phthalate; ^d^ MnOP—mono-n-octyl phthalate; ^e^ ∑DEHP—molar sum of the di (ethylhexyl)phthalate metabolites; ^f^ M/W: number of men/women.

**Table 2:** Characteristics of the sample.

	Men (N = 52)	Women (N = 60)	*p*-Value
Age (range)	40.1 (30–67)	37.8 (29–43)	0.016 *
BMI ^a^ (% of subjects)			
Normal	53.8	71.7	0.005 *
Overweight	36.5	13.3	
Obese	9.6	5.0	
Underweight	0.0	8.3	
Unknown	1.3	1.7	
Present smokers (%)	21.2	15.0	0.358
Previously smokers (%)	28.8	20.0	
Alcohol consumption			
Daily	19.2	3.3	0.011 *
Weekly	55.8	45.0	
Monthly	15.4	23.3	
Never	7.7	21.7	
Missing	1.9	6.7	
Residence area (%)			
Urban	67.3	81.7	0.388
Rural	17.3	10.0	
Cost	1.9	3.3	
Industrial	3.8	1.7	
Urban and industrial	1.9	0.0	
Other	5.7	1.7	
Missing	1.9	3.3	
Job type (%)			
Soldiers	9.6	0.0	0.000 *
Factory workers	23.1	1.7	
Teachers	1.9	16.7	
Employees, computer operators	44.2	48.3	
Healthcare professionals	5.8	11.7	
Cleaning workers/caterers	1.9	3.3	
Tradesman/sales clercks	0.0	5.0	
Artisan	3.8	0.0	
Unemployed	0.0	3.3	
Others	3.8	9.6	
Missing	5.8	10.0	
Infertility factor of the couple ^c^ (%)			
Ovulatory and endocrine dysfunctions	13.5	18.2	0.328
Endometriosis	3.8	5.0	
Reduced ovarian reserve	7.7	15.1	
RPL ^b^	7.6	10.0	
Idiopathic	26.9	24.9	
Male factor	23.3	6.7	
Reduced ovarian reserve + endometriosis	1.9	1.7	
Reduced ovarian reserve + RPL ^b^	0.0	1.7	
Tubal factor	0.0	5.0	
Male + female factors	11.5	10.0	
Others	3.8	1.7	

^a^ BMI: body mass index; ^b^ RPL: recurrent pregnancy loss; ^c^ the infertility factors are of the couple in which the single subject (male or female) is in * *t*-test or χ^2^ test significance *p* < 0.05.

**Table 3:** Results of urinary phthalate metabolites concentration for men in the two sub-groups.

µg/g Creatinine	MnBP ^a^	MEP ^b^	MBzP ^c^	MnOP ^d^	∑DEHP ^e^
>90th Percentile—Number of Subjects	12	9	9	14	12
Mean	120.55	1931.51	15.54	10.84	86.01
Median	106.94	880.20	12.63	8.36	72.02
Standard deviation	55.5	2173.00	7.34	6.01	47.23
Minimum	62.59	347.27	7.78	4.99	37.04
Maximun	245.13	7113.22	28.51	20.90	205.01
<10th Percentile—Number of Subjects	7	15	10	4	11
Mean	1.87	0.93	0.54	0.06	0.96
Median	1.63	1.05	0.60	0.05	0.92
Standard deviation	0.80	0.29	0.14	0.04	0.26
Minimum	0.91	0.11	0.18	0.03	0.70
Maximun	3.18	1.20	0.60	0.14	1.30

^a^ MnBP—mono-n-butyl phthalate; ^b^ MEP—monoethyl phthalate; ^c^ MBzP—monobenzyl phthalate; ^d^ MnOP—mono-n-octyl phthalate; ^e^ ∑DEHP—molar sum of the di (ethylhexyl)phthalate metabolites.

**Table 4:** Results of urinary phthalate metabolites concentration for women in the two sub-groups.

µg/g Creatinine	MnBP ^a^	MEP ^b^	MBzP ^c^	MnOP ^d^	∑DEHP ^e^
>90th Percentile—Number of Subjects	13	8	14	14	10
Mean	131.40	2549.59	36.09	9.81	79.14
Median	121.00	1049.14	28.26	8.75	63.00
Standard deviation	74.37	2735.39	26.18	3.92	46.93
Minimum	47.35	359.94	10.60	4.62	42.28
Maximun	281.71	7574.72	93.39	16.77	187.24
<10th Percentile—Number of Subjects	14	9	19	12	17
Mean	1.33	0.73	0.35	0.09	0.89
Median	1.25	0.71	0.33	0.08	1.10
Standard deviation	0.68	0.19	0.20	0.04	0.38
Minimum	0.10	0.55	0.07	0.02	0.10
Maximun	2.38	1.18	0.60	0.18	1.19

^a^ MnBP—mono-n-butyl phthalate; ^b^ MEP—monoethyl phthalate; ^c^ MBzP—monobenzyl phthalate; ^d^ MnOP—mono-n-octyl phthalate; ^e^ ∑DEHP—molar sum of the di (ethylhexyl) phthalate metabolites.

**Table 5:** Results of urinary concentrations of oxidative stress biomarkers for men.

N = 52 µg/g Creatinine	8-oxoGua ^1^	8-oxoGuo ^2^	8-oxodGuo ^3^	3-NO_2_Tyr ^4^	5-MeCyt ^5^
Mean	19.49	6.38	3.76	33.01	13.55
Median	14.94	4.75	2.58	23.18	9.58
Standard deviation	15.98	5.12	3.84	34.56	9.37
Minimum	1.21	1.44	0.07	5.60	3.73
Maximum	68.81	31.64	18.15	224.96	37.25
5th percentile	2.86	1.80	0.25	6.93	4.73
95th percentile	55.30	16.20	14.10	89.58	35.60

^1^ 8-oxoGua: 8-oxo-7,8-dihydroguanine; ^2^ 8-oxo-Guo: 8-Oxo-7,8-guanosine; ^3^ 8-oxodGuo: 8-Oxo-7,8-dihydro-2′-deoxyguanosine; ^4^ 3-NO_2_Tyr: 3-nitrotyrosine; ^5^ 5MeCyt: 5-methylcytidine.

**Table 6:** Results of urinary concentrations of oxidative stress biomarkers for women.

N = 60 µg/g Creatinine	8-oxoGua ^1^	8-oxoGuo ^2^	8-oxodGuo ^3^	3-NO_2_Tyr ^4^	5-MeCyt ^5^
Mean	17.84	4.46	3.89	19.73	13.79
Median	10.17	4.31	2.11	15.43	10.67
Standard deviation	21.13	2.42	4.31	18.05	13.53
Minimum	1.99	0.88	0.07	2.56	3.61
Maximum	111.18	10.37	17.57	117.18	95.83
5th percentile	2.79	1.13	0.22	3.61	4.08
95th percentile	62.70	9.03	13.26	55.58	34.10

^1^ 8-oxoGua: 8-oxo-7,8-dihydroguanine; ^2^ 8-oxo-Guo: 8-Oxo-7,8-guanosine; ^3^ 8-oxodGuo: 8-Oxo-7,8-dihydro-2′-deoxyguanosine; ^4^ 3-NO_2_Tyr: 3-nitrotyrosine; ^5^ 5-MeCyt: 5methylcytidine.

**Table 7:** *t*-test in the comparison of mean values of oxidized bases (logarithmic transformed) in the two sub-groups of high and low levels of phthalates metabolites in men and women.

	Mean Values (log_10_)		8-oxoGua	*p*	8-oxoGuo	*p*	8-oxodGuo	*p*	3-NO_2_Tyr	*p*	5-MeCyt	*p*
Men	MnOP	High	1.34	0.087	0.85	0.086	0.56	0.213	1.48	0.680	1.29	0.003 *
		Low	1.02		0.64		0.31		1.42		1.0	
	MEP	High	1.13	0.926	0.81	0.421	0.38	0.289	1.49	0.542	1.19	0.012 *
		Low	1.11		0.73		0.55		1.41		0.91	
	∑DEHP	High	1.25	0.104	0.79	0.005 *	0.40	0.007 *	1.46	0.000 *	1.24	0.000 *
		Low	0.81		0.34		−0.25		0.93		0.76	
	MBzP	High	1.36	0.097	0.85	0.044 *	0.50	0.237	1.56	0.004 *	1.26	0.102
		Low	1.09		0.61		0.21		1.15		1.03	
	MnBP	High	1.33	0.189	0.82	0.094	0.38	0.162	1.56	0.001 *	1.21	0.131
		Low	1.12		0.60		0.04		1.08		1.01	
Women	MnOP	High	1.24	0.751	0.68	0.414	0.19	0.026 *	1.37	0.662	1.37	0.038 *
		Low	1.19		0.78		0.65		1.31		0.22	
	MEP	High	0.94	0.855	0.71	0.044 *	0.48	0.211	1.34	0.051	1.14	0.097
		Low	0.97		0.47		0.18		1.09		0.89	
	∑DEHP	High	1.12	0.826	0.72	0.007 *	0.30	0.097	1.34	0.000 *	0.34	0.005 *
		Low	1.09		0.45		−0.06		0.89		0.17	
	MBzP	High	1.21	0.160	0.68	0.046 *	0.45	0.077	1.25	0.050	1.20	0.004 *
		Low	0.99		0.47		0.09		1.03		0.95	
	MnBP	High	1.23	0.252	0.80	0.001 *	0.54	0.208	1.45	0.003 *	1.25	0.000 *
		Low	1.03		0.51		0.25		1.11		0.92	

* significant value.

## Data Availability

The data presented in this study are available on request from the corresponding author. The data are not publicly available due to privacy and ethical reasons.

## Supplementary Materials

The following supporting information can be downloaded at: https://www.mdpi.com/article/10.3390/toxics10020061/s1, Table S1: Multiple linear regression data; Table S2: Concentrations of phthalates and biomarkers of oxidative stress: a comparison of published studies.

## References

[1] Hashemi M., Amin M.M., Chavoshani A., Rafiei N., Ebrahimpour K., Kelishadi R. (2021). Relationship of Urinary Phthalate Metabolites with Cardiometabolic Risk Factors and Oxidative Stress Markers in Children and Adolescents. J. Environ. Public Health.

[2] Rowdhwal S.S.S., Chen J. (2018). Toxic Effects of Di-2-ethylhexyl Phthalate: An Overview. BioMed Res. Int..

[3] Tranfo G., Caporossi L., Pigini D., Capanna S., Papaleo B., Paci E. (2018). Temporal Trends of Urinary Phthalate Concentrations in Two Populations: Effects of Reach Authorization after Five Years. Int. J. Environ. Res. Public Health.

[4] Tranfo G., Caporossi L., Paci E., Aragona C., Romanzi D., De Carolis C., De Rosa M., Capanna S., Papaleo B., Pera A. (2012). Urinary phthalate monoesters concentration in couples with infertility problems. Toxicol. Lett..

[5] Guo Y., Weck J., Sundaram R., Goldstone A.E., Louis G.B., Kannan K. (2014). Urinary Concentrations of Phthalates in Couples Planning Pregnancy and Its Association with 8-Hydroxy-2′-deoxyguanosine, a Biomarker of Oxidative Stress: Longitudinal Investigation of Fertility and the Environment Study. Environ. Sci. Technol..

[6] Rocha B.A., Asimakopoulos A.G., Barbosa F., Kannan K. (2017). Urinary concentrations of 25 phthalate metabolites in Brazilian children and their association with oxidative DNA damage. Sci. Total Environ..

[7] Agarwal A., Gupta S., Sharma R.K. (2005). Role of oxidative stress in female reproduction. Reprod. Biol. Endocrinol..

[8] Agarwal A., Virk G., Ong C., Du Plessis S.S. (2014). Effect of Oxidative Stress on Male Reproduction. World J. Men’s Health.

[9] Bisht S., Faiq M., Tolahunase M., Dada R. (2017). Oxidative stress and male infertility. Nat. Rev. Urol..

[10] Drevet J.R., Aitken R.J. (2020). Oxidation of Sperm Nucleus in Mammals: A Physiological Necessity to Some Extent with Adverse Impacts on Oocyte and Offspring. Antioxidants.

[11] Agarwal A., Aponte-Mellado A., Premkumar B.J., Shaman A., Gupta S. (2012). The effects of oxidative stress on female reproduction: A review. Reprod. Biol. Endocrinol..

[12] Martínez M.C., Andriantsitohaina R. (2009). Reactive Nitrogen Species: Molecular Mechanisms and Potential Significance in Health and Disease. Antioxid. Redox Signal..

[13] Aly H.A.A., Hassan M.H., A El-Beshbishy H.A., Alahdal A.M., Osman A.-M.M. (2015). Dibutyl phthalate induces oxidative stress and impairs spermatogenesis in adult rats. Toxicol. Ind. Health.

[14] Cruz D.F., Fardilha M. (2016). Relevance of peroxynitrite formation and 3-nitrotyrosine on spermatozoa physiology. Porto Biomed. J..

[15] Shono T., Taguchi T. (2013). Short-time exposure to mono-n-butyl phthalate (MBP)-induced oxidative stress associated with DNA damage and the atrophy of the testis in pubertal rats. Environ. Sci. Pollut. Res..

[16] Morielli T., O’Flaherty C. (2015). Oxidative stress impairs function and increases redox protein modifications in human spermatozoa. Reproduction.

[17] Cooke M.S., Olinski R., Loft S. (2008). Measurement and Meaning of Oxidatively Modified DNA Lesions in Urine. Cancer Epidemiol. Biomark. Prev..

[18] Matt S., Hofmann T.G. (2016). The DNA damage-induced cell death response: A roadmap to kill cancer cells. Cell. Mol. Life Sci..

[19] Gan W., Liu X.-L., Yu T., Zou Y.-G., Li T.-T., Wang S., Deng J., Wang L.-L., Cai J.-P. (2018). Urinary 8-oxo-7,8-dihydroguanosine as a Potential Biomarker of Aging. Front. Aging Neurosci..

[20] Guo C., Xie C., Chen Q., Cao X., Guo M., Zheng S., Wang Y. (2018). A novel malic acid-enhanced method for the analysis of 5-methyl-2′-deoxycytidine, 5-hydroxymethyl-2′-deoxycytidine, 5-methylcytidine and 5-hydroxymethylcytidine in human urine using hydrophilic interaction liquid chromatography-tandem mass spectrometry. Anal. Chim. Acta.

[21] Caporossi L., Alteri A., Campo G., Paci E., Tranfo G., Capanna S., Papaleo E., Pigini D., Viganò P., Papaleo B. (2020). Cross Sectional Study on Exposure to BPA and Phthalates and Semen Parameters in Men Attending a Fertility Center. Int. J. Environ. Res. Public Health.

[22] Caporossi L., Viganò P., Paci E., Capanna S., Alteri A., Campo G., Pigini D., De Rosa M., Tranfo G., Papaleo B. (2021). Female Reproductive Health and Exposure to Phthalates and Bisphenol A: A Cross Sectional Study. Toxics.

[23] Andreoli R., Manini P., De Palma G., Alinovi R., Goldoni M., Niessen W.M., Mutti A. (2010). Quantitative determination of urinary 8-oxo-7,8-dihydro-2′-deoxyguanosine, 8-oxo-7,8-dihydroguanine, 8-oxo-7,8-dihydroguanosine, and their non-oxidized forms: Daily concentration profile in healthy volunteers. Biomarkers.

[24] Kroll M.H., Chesler R., Hagengruber C., Blank D.W., Kestner J., Rawe M. (1986). Automated determination of urinary creatinine without sample dilution: Theory and practice. Clin. Chem..

[25] (2010). Putting gender on the agenda. Nature.

[26] Messing K., Dumais L., Courville J., Seifert A.M., Boucher M. (1994). Evaluation of exposure data from men and women with the same job title. J. Occup. Med..

[27] Franconi F., Brunelleschi S., Steardo L., Cuomo V. (2007). Gender differences in drug responses. Pharmacol. Res..

[28] Messing K., A Silverstein B. (2009). Gender and occupational health. Scand. J. Work. Environ. Health.

[29] Hlisníková H., Petrovičová I., Kolena B., Šidlovská M., Sirotkin A. (2020). Effects and Mechanisms of Phthalates’ Action on Reproductive Processes and Reproductive Health: A Literature Review. Int. J. Environ. Res. Public Health.

[30] Valko M., Leibfritz D., Moncol J., Cronin M.T.D., Mazur M., Telser J. (2007). Free radicals and antioxidants in normal physiological functions and human disease. Int. J. Biochem. Cell Biol..

[31] Tatone C., Amicarelli F., Carbone M.C., Monteleone P., Caserta D., Marci R., Artini P.G., Piomboni P., Focarelli R. (2008). Cellular and molecular aspects of ovarian follicle ageing. Hum. Reprod. Update.

[32] Agarwal A., Gupta S., Sekhon L., Shah R. (2008). Redox Considerations in Female Reproductive Function and Assisted Reproduction: From Molecular Mechanisms to Health Implications. Antioxid. Redox Signal..

[33] Matos L., Stevenson D., Gomes F., Silva-Carvalho J., Almeida H. (2009). Superoxide dismutase expression in human cumulus oophorus cells. Mol. Hum. Reprod..

[34] Devine P.J., Perreault S.D., Luderer U. (2012). Roles of Reactive Oxygen Species and Antioxidants in Ovarian Toxicity. Biol. Reprod..

[35] Gupta R., Schuh R., Fiskum G., Flaws J. (2006). Methoxychlor causes mitochondrial dysfunction and oxidative damage in the mouse ovary. Toxicol. Appl. Pharmacol..

[36] Minamiyama Y., Ichikawa H., Takemura S., Kusunoki H., Naito Y., Yoshikawa T. (2010). Generation of reactive oxygen species in sperms of rats as an earlier marker for evaluating the toxicity of endocrine-disrupting chemicals. Free Radic. Res..

[37] Botelho G.G.K., Bufalo A.C., Boareto A.C., Muller J.C., Morais R.N., Martino-Andrade A.J., Lemos K.R., Dalsenter P.R. (2009). Vitamin C and Resveratrol Supplementation to Rat Dams Treated with Di(2-ethylhexyl)phthalate: Impact on Reproductive and Oxidative Stress End Points in Male Offspring. Arch. Environ. Contam. Toxicol..

[38] Erkekoglu P., Rachidi W., Yuzugullu O.G., Giray B., Favier A., Ozturk M., Hincal F. (2010). Evaluation of cytotoxicity and oxidative DNA damaging effects of di(2-ethylhexyl)-phthalate (DEHP) and mono(2-ethylhexyl)-phthalate (MEHP) on MA-10 Leydig cells and protection by selenium. Toxicol. Appl. Pharmacol..

[39] Asghari M.H., Saeidnia S., Abdollahi M. (2015). A Review on the Biochemical and Molecular Mechanisms of Phthalate-Induced Toxicity in Various Organs with a Focus on the Reproductive System. Int. J. Pharmacol..

[40] Zhang Y.J., Wu L.H., Wang F., Liu L.Y., Zeng E.Y., Guo Y. (2021). DNA oxidative damage in pregnant women upon exposure to conventional and alternative phthalates. Environ. Int..

[41] Cathey A.L., Eaton J.L., Ashrap P., Watkins D.J., Rosario Z.Y., Vega C.V., Alshawabkeh A.N., Cordero J.F., Mukherjee B., Meeker J.D. (2021). Individual and joint effects of phthalate metabolites on biomarkers of oxidative stress among pregnant women in Puerto Rico. Environ. Int..

[42] Ferguson K., McElrath T.F., Chen Y.-H., Mukherjee B., Meeker J.D. (2015). Urinary Phthalate Metabolites and Biomarkers of Oxidative Stress in Pregnant Women: A Repeated Measures Analysis. Environ. Health Perspect..

[43] Lin C.-Y., Chen P.-C., Hsieh C.-J., Chen C.-Y., Hu A., Sung F.-C., Lee H.-L., Su T.-C. (2017). Positive Association between Urinary Concentration of Phthalate Metabolites and Oxidation of DNA and Lipid in Adolescents and Young Adults. Sci. Rep..

[44] Ferguson K.K., Cantonwine D.E., Rivera-González L.O., Loch-Caruso R., Mukherjee B., Del Toro L.V.A., Jiménez-Vélez B., Calafat A.M., Ye X., Alshawabkeh A.N. (2014). Urinary Phthalate Metabolite Associations with Biomarkers of Inflammation and Oxidative Stress Across Pregnancy in Puerto Rico. Environ. Sci. Technol..

[45] Ferguson K.K., McElrath T.F., Mukherjee B., Loch-Caruso R., Meeker J.D. (2015). Associations between Maternal Biomarkers of Phthalate Exposure and Inflammation Using Repeated Measurements across Pregnancy. PLoS ONE.

[46] Ferguson K., McElrath T.F., Chen Y.-H., Loch-Caruso R., Mukherjee B., Meeker J.D. (2015). Repeated measures of urinary oxidative stress biomarkers during pregnancy and preterm birth. Am. J. Obstet. Gynecol..

[47] Stein T.P., Scholl T.O., Schluter M.D., Leskiw M.J., Chen X., Spur B.W., Rodriguez A. (2008). Oxidative stress early in pregnancy and pregnancy outcome. Free Radic. Res..

[48] Liu C., Duan P., Chen Y.-J., Deng Y.-L., Luo Q., Miao Y., Cui S.-H., Liu E.-N., Wang Q., Wang L. (2019). Mediation of the relationship between phthalate exposure and semen quality by oxidative stress among 1034 reproductive-aged Chinese men. Environ. Res..

[49] Waits A., Chen H.-C., Kuo P.-L., Wang C.-W., Huang H.-B., Chang W.-H., Shih S.-F., Huang P.-C. (2020). Urinary phthalate metabolites are associated with biomarkers of DNA damage and lipid peroxidation in pregnant women—Tainan Birth Cohort Study (TBCS). Environ. Res..

[50] Chang W.-H., Tsai Y.-S., Wang J.-Y., Chen H.-L., Yang W.-H., Lee C.-C. (2019). Sex hormones and oxidative stress mediated phthalate-induced effects in prostatic enlargement. Environ. Int..

[51] Tranfo G., Paci E., Carrieri M., Marchetti E., Sisto R., Gherardi M., Costabile F., Bauleo L., Ancona C., Pigini D. (2019). Levels of Urinary Biomarkers of Oxidatively Generated Damage to DNA and RNA in Different Groups of Workers Compared to General Population. Int. J. Environ. Res. Public Health.

[52] Yuan X.-Q., Du Y.-Y., Liu C., Guo N., Teng X.-M., Hua X., Yao Y.-C., Deng Y.-L., Zeng Q., Deng T.-R. (2020). Phthalate metabolites and biomarkers of oxidative stress in the follicular fluid of women undergoing in vitro fertilization. Sci. Total Environ..

[53] Chen K.M., Calcagnotto A., Zhu J., Sun Y., Wan E.B., Karam J.J. (2018). Comparison of an HPLC-MS/MS Method with Multiple Commercial ELISA Kits on the Determination of Levels of 8-oxo-7,8-Dihydro-2′-Deoxyguanosine in Human Urine. J. New Dev. Chem..

[54] Hueiwang A.J., Mu R.C., Ruei N.L., Chih H.P., Wen Y.L. (2015). Measurement of 8-Oxo-7,8-Dihydro-2′-Deoxyguanosine in Human Semen and Urine by Isotope-Dilution Liquid Chromatography-Tandem Mass Spectrometry with On-Line Solid-Phase Extraction: Comparison with a Commercial Available Enzyme-Linked Immunosorbent Assay. Andrology.

[55] Barregard L., Møller P., Henriksen T., Mistry V., Koppen G., Rossner P., Sram R.J., Weimann A., Poulsen H.E., Nataf R. (2013). Human and Methodological Sources of Variability in the Measurement of Urinary 8-Oxo-7,8-dihydro-2′-deoxyguanosine. Antioxid. Redox Signal..

[56] Ambe K., Sakakibara Y., Sakabe A., Makino H., Ochibe T., Tohkin M. (2019). Comparison of the developmental/reproductive toxicity and hepatotoxicity of phthalate esters in rats using an open toxicity data source. J. Toxicol. Sci..

