# Supplementary material for: Phthalate Exposure and Biomarkers of Oxidation of Nucleic Acids: Results on Couples Attending a Fertility Center
Source: Toxics. 2022 Jan 29;10(2):61. doi: 10.3390/toxics10020061 (PMC8876283; doi:10.3390/toxics10020061)
Supplement: Supplementary file 1 [file toxics-10-00061-s001.zip › toxics-1542639 resubmitted proof supplementary.pdf]

toxics

MDPI

Supplementary Materials: Phthalate Exposure and Biomarkers of Oxidation of Nucleic Acids: Results on Couples Attending a Fertility Center

Daniela Pigini, Lidia Caporossi, Enrico Paci, Silvia Capanna, Paola Viganò, Alessandra Alteri, Elisa Rabellotti, Flavia Buonaurio, Bruno Papaleo and Giovanna Tranfo

Table S1. Multiple linear regression data.

Women group: Bases and phthalates logarithmic (log 10) transformed	*p*
oxoGua = 0.633+0.021 (age) − 0.018 (BMI) − 0.306(smoke) + 0.019(alcohol) + 0.325(MnBP)-0.168(MEP)* + 0.083(MBzP) − 0.016(MnOP) + 0.072(DEHP) − 0.086(BisfenoloA)	* $p = 0.019$
oxoGuo = 0.376 + 0.006(age) + 0.004(BMI) − 0.157(smoke) − 0.033(alcohol) + 0.123(MnBP) − 0.032(MEP) + 0.029(MBzP) − 0.096(MnOP) + 0.061(DEHP) − 0.038(BisfenoloA)	$p > 0.05$
oxodGuo = 0.528 + 0,045(age) − 0.007(BMI) − 0.599(smoke) + 0.047(alcohol) − 0.099(MnBP) + 0.043(MEP) − 0.002(MBzP) − 0.252(MnOP)* + 0.001(DEHP) + 0.111(BisfenoloA)	*$p = 0.031$
NO2Tyr = 0.983 − 0.007(age) − 0.010(BMI) − 0.425(smoke) + 0.118(alcohol) + 0.127(MnBP) − 0.022(MEP) − 0.069(MBzP) + 0.007(MnOP) + 0.143(DEHP) − 0.061(BisfenoloA)	$p > 0.05$
MeCyt = 0.600 − 0,013(age) − 0.009(BMI) + 0.066(smoke) − 0.018(alcohol) + 0.176(MnBP) − 0.047(MEP) + 0.128(MBzP) + 0.021(MnOP) + 0.125(DEHP) − 0.028(BisfenoloA)	$p > 0.05$

Men group: Bases and phthalates logarithmic (log 10) transformed	
oxoGua = 1.293 + 0.021(age) − 0.018(BMI) − 0.0306(smoke) + 0.019(alcohol) − 0.347(MnBP) + 0.004(MEP) + 0.515(MBzP)* + 0.079(MnOP) + 0.084(DEHP) + 0.044(BisfenoloA)	*$p = 0.011$
oxoGuo = 0.779 + 0.006(age) + 0.004(BMI) − 0.157(smoke) − 0.033(alcohol) − 0.076(MnBP) − 0.013(MEP) + 0.219(MBzP) + 0.066(MnOP) + 0.016(DEHP) + 0.061(BisfenoloA)	$p > 0.05$
oxodGuo = 0.708 + 0.045(age) − 0.007(BMI) − 0.599(smoke) + 0.047(alcohol) − 0.327(MnBP) − 0.002(MEP) + 0.124(MBzP) + 0.072(MnOP) + 0.194(DEHP) + 0.143(BisfenoloA)	$p > 0.05$
NO2Tyr = 1.192 -0.007(age) − 0.010(BMI) − 0.425(smoke) + 0.118(alcohol) + 0.025(MnBP) − 0.058(MEP) + 0.197(MBzP) + 0.015(MnOP) + 0.136(DEHP) − 0.022(BisfenoloA)	$p > 0.05$
MeCyt = 0.994 − 0.013(age) − 0.009(BMI) + 0.066(smoke) − 0.018(alcohol) − 0.087(MnBP) + 0.036(MEP) + 0.226(MBzP)* + 0.045(MnOP) + 0.106(DEHP) + 0.138(BisfenoloA)*	*$p = 0.035$; $p = 0.043$

*Significant data with the corresponding *p* value

Table S2. Concentrations of phthalates and biomarkers of oxidative stress: a comparison of published studies.

Reference	analytical method	subjects	Biomarkers of oxidative stress (urine)	Urinary phthalate metabolites
[41]	ESI-MS/MS[1]	364 pregnant women	Median (μg/g creatinine) 8-OHdG[2] = 10.7	Median (μg/g creatinine): MEP[3] = 8.17 MBzP[4] = nd, MnBP[5] = 51.8, ΣDEHP[6] = 17.8
[43]	Enzyme immunoassay HPLC-MS/MS[7]	Multiple time points of pregnant women	Median (μg/L) 8-OHdG = 130	Median (μg/L): MEP = 121, MBzP = 6.38, MnBP = 16.5 ΣDEHP = 0.31 μmoli/L
[44]	HPLC/MS/MS ESI-MS/MS	469 couples	Median (μg/g creatinine): 8-OHdG = 3.13	Median (μg/g creatinine): MMP[8] = 0.48, MEP = 85.2, MCPP[9] = 4.50, MnBP = 7.66, MiBP = 4.36 MBzP = 3.80, ΣDEHP = 54.8 Σ14phthalates = 249
[45]	ESI-MS/MS	300 children	Median (μg/L): 8-OHdG = 2.80	Median (μg/L): MEP = 57.3, MBzP = 1.91, ΣDEHP = 110
[46]	HPLC/MS/MS	751 students	Geometric means (μg/g creatinine): 8-OHdG = 2.02	Geometric means (μg/g Creatinine): MEP = 31.21 MnBP = 35.52 MBzP = 1.93 MMP = 7.51 MiBP[10] = 15.09 ΣDEHP = 0.22 μmoli/g creatinine
[52]	HPLC/MS/MS	1034 men	Median (μg/L): 8-OHdG = 8.2	Median (μg/L): MEP = 18, MBzP = 2.9, MBP = 63, MnOP[11] = 0.12

[1] ESI-MS/MS: Electrospray Ionization tandem mass spectrometry; [2]8OHdG: 8-Oxo-2'-desossiguanosina; [3]MEP- monoethyl phthalate; [4]MBzP- monobenzyl phthalate; [5]MnBP- mono-n-butyl phthalate; [6]∑DEHP- molar sum of the di (ethylhexyl) phthalate metabolites; [7] HPLC-MS/MS: high performance liquid chromatography/tandem mass spectrometry; [8]MMP: Monomethyl phthalate; [9]MCPP: mono-(3-carboxypropyl) phthalate; [10]MiBP: mono isobutylphthalate; [11]MnOP: mono-n-octyl phthalate.
